# Supplementary material for: A high-resolution mRNA expression time course of embryonic development in zebrafish
Source: eLife. 2017 Nov 16;6:e30860. doi: 10.7554/eLife.30860 (PMC5690287; doi:10.7554/eLife.30860)
Supplement: Supplementary file 6. [file elife-30860-supp6.zip › biolayout-clusters-files/Cluster163-genes.html]

Cluster163


# Cluster163: Genes

| Ensembl ID | Gene Name | Chr | Start | End | Biotype |
| --- | --- | --- | --- | --- | --- |
| ENSDARG00000063525 | adpgk | 25 | 2553332 | 2570512 | protein\_coding |
| ENSDARG00000020679 | clpp | 3 | 33999997 | 34007270 | protein\_coding |
| ENSDARG00000008239 | mocs3 | 17 | 42536859 | 42567457 | protein\_coding |
| ENSDARG00000075530 | pigu | 6 | 50711841 | 50731557 | protein\_coding |
| ENSDARG00000100057 | ppfibp1b | 18 | 15500495 | 15582944 | protein\_coding |
| ENSDARG00000052721 | prim2 | 13 | 23357832 | 23481919 | protein\_coding |
| ENSDARG00000040861 | tcf25 | 18 | 31138891 | 31228326 | protein\_coding |
| ENSDARG00000025754 | ube2f | 1 | 5948542 | 5966136 | protein\_coding |
